# SLIT3: a novel regulator of odontogenic differentiation through Akt/GSK3β/β-catenin signaling pathway

**DOI:** 10.1038/s41368-026-00426-7

**Published:** 2026-04-13

**Authors:** Lingyu Jiang, Liu Liu, Fan Yang, Yujia Cui, Jing Xie, Dongzhe Song, Yi Fan, Dingming Huang, Jianxun Sun

**Affiliations:** 1 State Key Laboratory of Oral Diseases & National Center for Stomatology & National Clinical Research Center for Oral Diseases, West China Hospital of Stomatology, Sichuan University, Chengdu, China; 2 Department of Stomatology, Nanfang Hospital, Southern Medical University, Guangzhou, China; 3 Department of Conservative Dentistry and Endodontics, West China Hospital of Stomatology, Sichuan University, Chengdu, China; 4 Centre for Craniofacial and Regenerative Biology, Faculty of Dentistry, Oral & Craniofacial Sciences, King’s College London, London, UK

**Keywords:** Molecular medicine, Molecular biology

## Abstract

The odontogenic differentiation of Stem Cells from Apical Papilla (SCAP) are governed by various extracellular matrix proteins, playing a crucial role in dentin formation and regeneration. Extracellular matrix protein SLIT3, a classical axon guidance molecule, has been identified as a clastokine linking bone resorption to formation. However, its role in odontogenesis is not well-documented. Thus, our study aimed to explore the effects and mechanisms of SLIT3 on SCAP proliferation and differentiation. Analysis of developing mouse molars showed that while *Slit3* mRNA was restricted to the dental mesenchyme, the SLIT3 protein was prominently detected on both odontoblasts and adjacent epithelial ameloblasts. Real time polymerase chain reaction (RT-PCR) and Western blot assays confirmed increased SLIT3 expression during SCAP odontogenic differentiation. SLIT3 siRNA knockdown and recombinant human SLIT3 (rhSLIT3) protein treatments were administered to SCAP. Cell Counting Kit-8 (CCK8) assays indicated that SLIT3 promotes SCAP proliferation, while alkaline phosphatase (ALP) and Alizarin red staining showed increased mineralization. Odontogenic markers DMP-1 and DSPP were also modulated accordingly. Additionally, rhSLIT3 treatment enhanced p-Akt and p-GSK3β levels in SCAP, promoting β-catenin nuclear translocation. The effects of SLIT3 were negated with an Akt/GSK3β/β-catenin signaling pathway inhibitor. Collectively, our data suggest that SLIT3 promotes SCAP proliferation and odontogenic differentiation via the Akt/GSK3β/β-catenin signaling pathway activation.

## Introduction

Pulp infection, necrosis, or periapical inflammation due to trauma or caries frequently affect immature permanent teeth, hindering root development. These teeth have short roots and thin dentin walls, lacking an apical stop, which increases the risk of root fracture.^1–3^ Thus, promoting root development in teeth with pulp necrosis is a desirable treatment approach. It is essential to examine and enhance the signaling molecules and mechanisms regulating tooth root development.

Root development follows crown formation, initiated by Hertwig’s epithelial root sheath.^4,5^ Mesenchymal stem cells in the tooth germ receive developmental signals from the sheath, possessing multi-directional differentiation potential. These cells can differentiate into odontoblasts, dental pulp cells, cementoblasts, and periodontal ligament cells, all crucial for root development.^6,7^

Stem cells from Apical Papilla (SCAP), derived from the apical papilla tissue of immature permanent teeth, were first isolated and cultured by Sonoyama et al.^8^ SCAP are considered the precursor to odontoblasts, elongating and polarizing under the influence of the epithelial root sheath to secrete and mineralize dentin.^9^ Due to their significant proliferation ability and differentiation potential, SCAP are frequently used as an ideal cell model in laboratory studies focused on tooth development and odontoblast differentiation.^10,11^

As structural proteins, extracellular matrix proteins interact with receptors and integrins on cell membranes, regulating growth factor and protease activities, which are crucial for dentin formation and mineralization.^12,13^ Recently, SLIT3, a member of the extracellular matrix protein SLIT family, has been implicated in numerous biological processes, such as angiogenesis, tumorigenesis, inflammation, and bone metabolic balance.^14–16^ SLIT3 acts as a novel bone coupling factor, promoting bone formation and inhibiting resorption in bone metabolic homeostasis.^17^ Although bone and dentin are distinct mineralized tissues, they share similarities in mineralization processes. However, research on SLIT3’s role in tooth development remains limited.

The mechanisms driving SLIT3’s potential role in odontogenesis are largely unexplored. A key candidate pathway is the Akt/GSK3β/β-catenin axis, a positive regulator of SCAP proliferation and odontogenic differentiation.^18,19^ This pathway, in turn, is classically regulated by the kinase Akt, which can phosphorylate and inactivate GSK3β, thereby preventing β-catenin degradation.^20^ Interestingly, SLIT/ROBO signaling, particularly through SLIT2, a homolog of SLIT3, directly influences Akt activity. This suggests a possible SLIT3-Akt-GSK3β-β-catenin signaling cascade in SCAP. Consequently, this study aims to investigate SLIT3’s effects on SCAP odontogenic differentiation and evaluate the specific hypothesis that SLIT3 activates the Akt/GSK3β/β-catenin signaling pathway.

The objective of this study was to examine SLIT3’s spatiotemporal expression during tooth development in vivo and in vitro, assessing its impact on SCAP proliferation and odontogenic differentiation. The role of Akt/GSK3β/β-catenin signaling pathway in the effect of SLIT3 on odontogenic differentiation of SCAP were also evaluated.

## Results

### Defining dental mesenchyme subgroups at different developmental stages in the integrated single-cell transcriptomic atlas

**Fig. 1:**
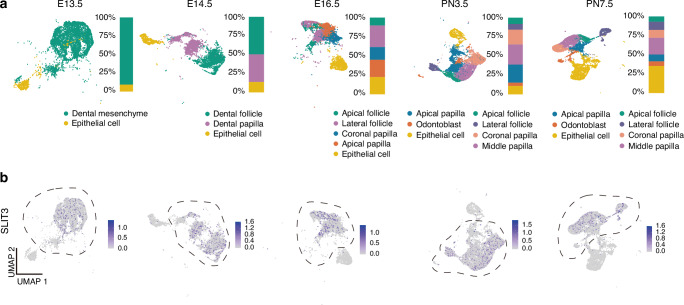
SLIT3 displays dynamic expression patterns across embryonic to postnatal tooth development. **a** UMAP plots illustrating single-cell data from embryos to postnatal stages in the tooth and surrounding tissues; **b** Expression of SLIT3 at different embryonic and postnatal stages, with dotted lines indicating mesenchymal cell subpopulations

Data cleaning and clustering analyses were conducted on GSE189381 in a manner similar to that described in a previously published study on cranial neural crest cells.^21^ The single-cell RNA sequencing data underwent preprocessing, including quality control, integration, principal component analysis, and dimensionality reduction. UMAP plots (Fig. 1a) visualized single-cell data from E13.5 to PN7.5 in dental and surrounding tissues. Subclustering analyses, alongside known markers for dental mesenchyme, identified dental mesenchyme subclusters marked by dotted frames (Fig. 1a, b).

At E13.5, early tooth development stages revealed two primary clusters: dental mesenchyme and epithelial cell clusters. By E14.5, distinct clusters of dental follicle and dental papilla cells emerged, indicating lineage segregation. At E16.5, the dental follicle differentiated into lateral and apical follicles, while dental papilla split into coronal and apical papillae. By PN3.5, approaching root development onset, follicle differentiation mirrored patterns at E16.5, and papilla subclusters comprised coronal, middle, apical papillae, and odontoblast clusters. At PN7.5, cell populations resembled those at PN3.5, suggesting completion of tooth root development. Notably, *Slit3* mRNA maintained consistent expression in dental mesenchyme subclusters with minimal to no expression in epithelial cell subclusters (Fig. 1b).

### SLIT3 is continuously expressed in odontoblasts of developing mandibular first molar in mice

**Fig. 2:**
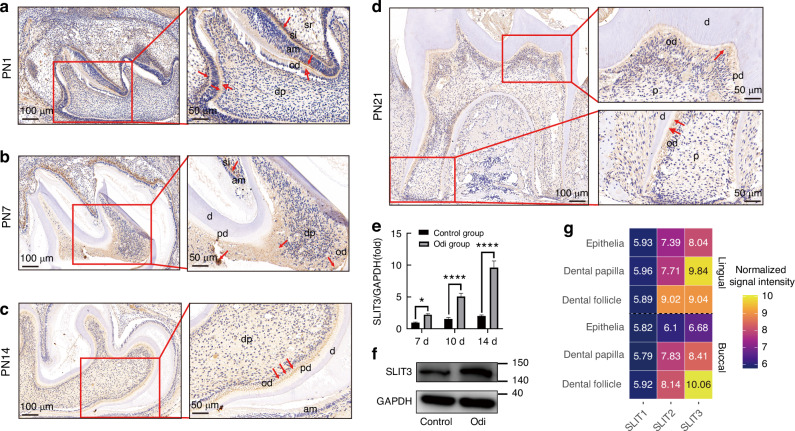
SLIT3 expression progressively increases during in vivo odontoblast maturation and in vitro odontogenic differentiation of SCAP. **a** Immunohistochemical staining results of mandibular first molar sections from PN1 mouse (20×, scale bar: 100 μm), and magnified immunohistochemical staining results of mandibular first molar sections from PN1 mouse (40×, scale bar: 50 μm); **b** Immunohistochemical staining results of mandibular first molar sections from PN7 mouse (20×, scale bar: 100 μm), and magnified immunohistochemical staining results of mandibular first molar sections from PN7 mouse (40×, scale bar: 50 μm); **c** Immunohistochemical staining results of mandibular first molar sections from PN14 mouse (20×, scale bar: 100 μm), and magnified immunohistochemical staining results of mandibular first molar sections from PN14 mouse (40×, scale bar: 50 μm); **d** Immunohistochemical staining results of mandibular first molar sections from PN21 mouse(20×, scale bar: 100 μm), and magnified immunohistochemical staining results of crown of mandibular first molar sections from PN21 mouse (40×, scale bar: 50 μm), and magnified immunohistochemical staining results of root of mandibular first molar sections from PN21 mouse (40×, scale bars: 50 μm); **e** Result of RT-PCR showed that the mRNA expression of SLIT3 was increased in the SCAP differentiated into odontoblasts. *P < 0.05, ****P < 0.000 1 versus the control group using two-way ANOVA followed by the Holm-Sidak post hoc multiple comparison test. **f** Result of Western blot showed that the protein level of SLIT3 was increased in the SCAP differentiated into odontoblasts at 10 days; **g** Expression patterns of SLIT1, SLIT2, and SLIT3 were reanalyzed using our laser capture microdissection-based microarray dataset, which profiles dental epithelium, dental papilla, and dental follicle tissues during mouse root development. E embryo, PN postnatal, Sr star reticular layer, Si middle layer, Am ameloblast, Od odontoblast, Dp dental papilla, D Dentin, Pd pre-dentine, P Pulp

Immunohistochemical staining of paraffin sections from the first mandibular molar tissues of PN1 mice showed SLIT3 expression in odontoblasts, ameloblasts, and the middle layer (Fig. 2a). On PN7, SLIT3 expression was observed in the dental papilla, odontoblasts, ameloblasts, and middle layer (Fig. 2b). In PN14 mice, SLIT3 was expressed in the odontoblast layer (Fig. 2c) but not elsewhere. By PN21, SLIT3 was expressed in both crown and root odontoblasts, with stronger expression in root odontoblasts (Fig. 2c, d). These findings suggest SLIT3’s significant role in odontoblast differentiation and maturation, and hard tissue formation. The strong protein signal in epithelial-derived ameloblasts contrasts with scRNA-seq and microarray data, which localize *Slit3* mRNA expression to the dental mesenchyme, indicating a paracrine action of the SLIT3 protein.

### Increased SLIT3 gene and protein expression during SCAP odontogenic differentiation

SCAP were isolated from an undeveloped, caries-free third molar. During mineralization medium-induced odontogenic differentiation of SCAP, RT-PCR results demonstrated a significant increase in Slit3 expression in the experimental group compared to the control group (*P* < 0.05), with the difference becoming more pronounced over time (*P* < 0.000 1) (Fig. 2e). Western blot analysis also showed higher SLIT3 protein expression in the experimental group during SCAP odontogenic differentiation (Fig. 2f). Our re-analysis of an independent microarray dataset from previous work, which distinguished anatomical regions, revealed high *Slit3* expression in the buccal dental follicle and lingual dental papilla groups, and lower expression in the buccal and lingual dental epithelial groups. Similarly, *Slit2* showed a similar expression pattern, albeit at lower levels than *Slit3* (Fig. 2g).

### SLIT3 enhances SCAP proliferation

SCAP exhibiting low and high SLIT3 expression were modeled in vitro. RT-PCR and Western blot analyses demonstrated significantly lower SLIT3 expression in the siSLIT3 group compared to the siNC group (Supplementary Fig. S1a–c), whereas the group treated with recombinant human SLIT3 (rhSLIT3) exhibited significantly higher SLIT3 expression than the control group. This confirms the suitability of SCAP with variable SLIT3 expression for subsequent experimental analyses.

Using the CCK8 kit, we assessed the effect of SLIT3 expression on SCAP proliferation. The results indicated that increased SLIT3 expression enhanced SCAP proliferation, whereas reduced SLIT3 expression diminished it (Supplementary Fig. S1d). These findings confirm that SLIT3 promotes SCAP proliferation.

### SLIT3 promotes SCAP mineralization and upregulates DMP-1 and DSPP expression

**Fig. 3:**
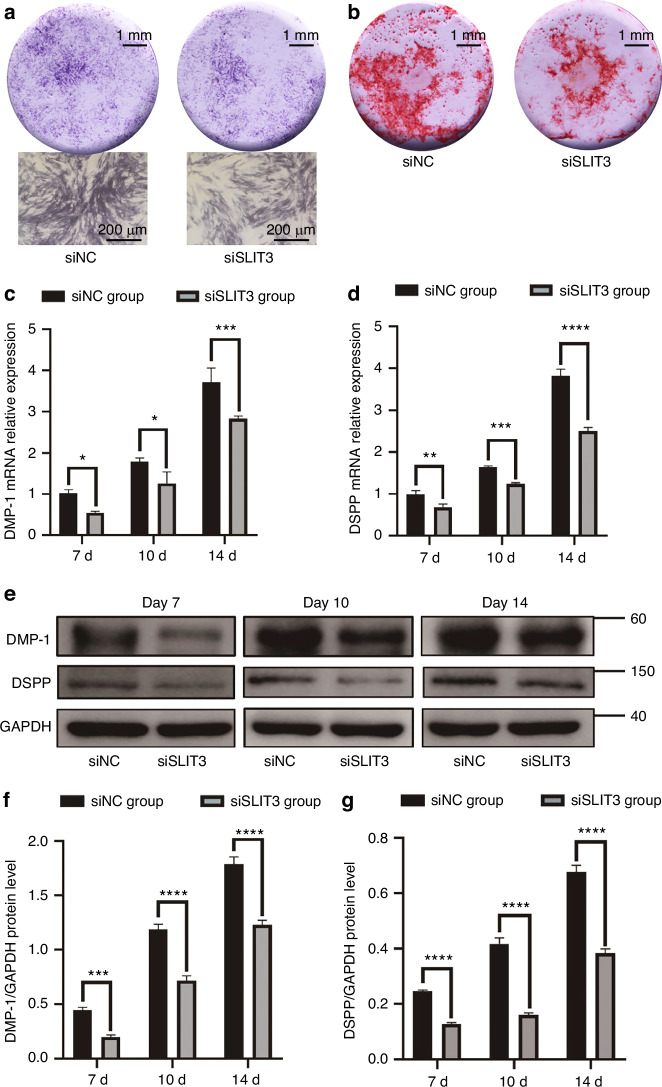
siRNA knockdown of SLIT3 inhibits the odontogenic differentiation of SCAP. **a** Representative images of alkaline phosphatase (ALP) staining in negative control group and SLIT3 siRNA group of SCAP after 7 days of odontogenic induction. Scale bars of images of stereomicroscope: 1 mm. Scale bars of images of optical microscope: 200 μm. **b** Representative images of alizarin red S (ARS) staining in negative control group (siNC) and SLIT3 siRNA group (siSLIT3) of SCAP after 14 days of odontogenic induction. Scale bars: 1 mm. **c**, **d** RT-PCR analysis of DMP-1 and DSPP mRNA expression in siNC and siSLIT3 groups after 7, 10, and 14 days of odontogenic induction. GAPDH was used as an internal control. *P < 0.05, **P < 0.01, ***P < 0.001, ****P < 0.000 1 versus the siNC group using two-way ANOVA followed by the Holm-Sidak post hoc multiple comparison test. **e** Western blot analysis of DMP-1 and DSPP expression in siNC and siSLIT3 groups after 7, 10 and 14 days of odontogenic induction. GAPDH normalized relative protein levels; **f**, **g** Quantitative analysis of relative protein levels of DMP-1 and DSPP in siNC and siSLIT3 groups. ***P < 0.001, ****P < 0.000 1 versus the siNC group using two-way ANOVA followed by the Holm-Sidak post hoc multiple comparison test

In SCAP with low SLIT3 expression, after 7 days of mineralization induction, ALP staining showed a lighter staining intensity in the siSLIT3 group compared to the siNC group (Fig. 3a). After 14 days, ARS staining indicated fewer mineralized nodules in the siSLIT3 group than in the siNC group (Fig. 3b), suggesting that reduced SLIT3 expression inhibited SCAP odontogenic differentiation.

**Fig. 4:**
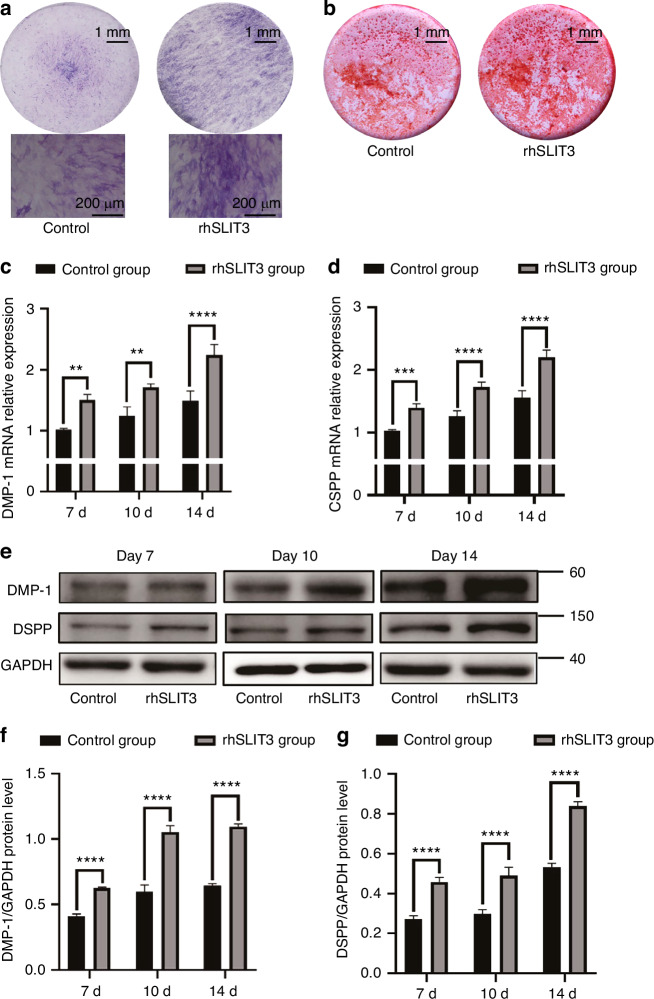
rhSLIT3 treatment enhances the odontogenic differentiation of SCAP. **a** Representative images of ALP staining in control group and recombinant human SLIT3 (rhSLIT3) group of SCAP after 7 days of odontogenic induction. Scale bars of images of stereomicroscope: 1 mm. Scale bars of images of optical microscope: 200 μm. **b** Representative images of ARS staining in control group and rhSLIT3 group of SCAP after 14 days of odontogenic induction. Scale bars: 1 mm. **c**, **d** RT-PCR analysis of DMP-1 and DSPP mRNA expression in control group and rhSLIT3 group after 7, 10, and 14 days of odontogenic induction. GAPDH was used as an internal control. **P < 0.01, ***P < 0.001, ****P < 0.000 1 versus the control group using two-way ANOVA followed by the Holm-Sidak post hoc multiple comparison test. **e** Western blot analysis of DMP-1 and DSPP expression in control group and rhSLIT3 group after 7, 10 and 14 days of odontogenic induction. GAPDH normalized relative protein levels; **f**, **g** Quantitative analysis of relative protein levels of DMP-1 and DSPP in control group and rhSLIT3 group. ****P < 0.000 1 versus the control group two-way ANOVA followed by the Holm-Sidak post hoc multiple comparison test

In contrast, SCAP treated with rhSLIT3 showed greater mineralization after 7 days of induction, as evidenced by ALP staining (Fig. 4a), and more extensive mineralization after 14 days, as revealed by ARS staining (Fig. 4b). This indicates that increased SLIT3 expression promoted SCAP odontogenic differentiation.

To further confirm SLIT3’s role in promoting SCAP odontogenic differentiation, RT-PCR and Western blot analyses were conducted to assess dentin formation markers. SCAP samples with low or high SLIT3 expression were collected on days 7, 10, and 14 post-mineralization induction. RT-PCR results showed decreased DMP-1 and DSPP expression in the siRNA group, whereas their expression levels increased in the rhSLIT3 group (Fig. 3c, d and Fig. 4c, d). Similarly, Western blot results demonstrated decreased protein expression of DMP-1 and DSPP in the siRNA group and increased expression in the rhSLIT3 group over the same period (Fig. 3e–g and Fig. 4e–g).

**Fig. 5:**
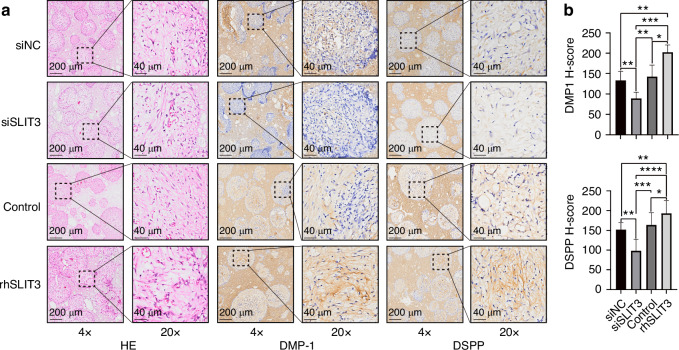
SLIT3 can promote odontogenic differentiation of SCAP in an in vivo ectopic transplantation model. **a** Representative images of Hematoxylin-Eosin staining and immunohistochemical staining for DMP-1 and DSPP in SLIT3-treated SCAP/β-TCP transplanted into nude mice. Scale bars of 4× images: 200 μm. Scale bars of 20× images: 40 μm. **b** Quantitative analysis of (**a**) using the H-score. *P < 0.05, **P < 0.01, ***P < 0.001, ****P < 0.000 1 versus the control group using one-way ANOVA followed by the Holm-Sidak post hoc multiple comparison test

In vivo, SLIT3 overexpression or knockdown in SCAP/Beta-tricalcium phosphate (β-TCP) complexes was further validated through ectopic transplantation into nude mice. Immunohistochemistry quantification revealed that SLIT3 overexpression enhanced SCAP odontogenic differentiation and increased DMP-1 and DSPP protein expression (Fig. 5a, b). Conversely, SLIT3 knockdown via siRNA reduced SCAP odontogenic differentiation and decreased DMP-1 and DSPP protein expression (Fig. 5a, b).

### SLIT3 activates the Akt/GSK3β/β-catenin signaling pathway, promoting the odontogenic differentiation of SCAP via ROBO2/ROBO3

**Fig. 6:**
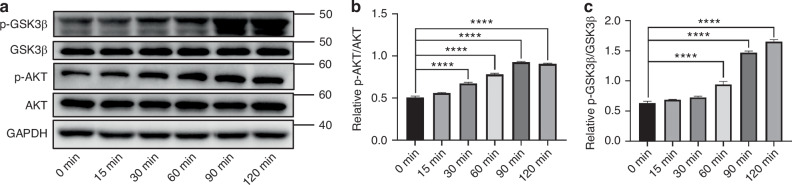
SLIT3 can promote the phosphorylation of Akt and GSK-3β in SCAP. **a** Western blot analysis of phosphorylation levels of Akt and GSK3β in SCAP after the addition of rhSLIT3. GAPDH normalized relative protein levels. **b**, **c** Quantitative analysis of p-Akt/Akt and p-GSK3β/GSK3β ratio in SCAP after the addition of rhSLIT3. ****P < 0.000 1 versus the 0 min group (untreated baseline control) using one-way ANOVA followed by the Holm-Sidak post hoc multiple comparison test

To elucidate the molecular mechanism underlying the pro-odontogenic effects of SLIT3, we first investigated whether it engages the Akt/GSK3β/β-catenin signaling module. We first hypothesized that SLIT3 could alter the activity of Akt and its well-established downstream target, GSK3β. To test this, SCAP were treated with rhSLIT3 over a time course. Western blot analysis revealed a rapid and significant increase in the phosphorylation of both Akt and GSK3β, evident within 30–60 min of stimulation (Fig. 6a–c). This result demonstrates that SLIT3 stimulation leads to the coordinated phosphorylation of Akt and inhibitory phosphorylation of GSK3β in SCAP.

**Fig. 7:**
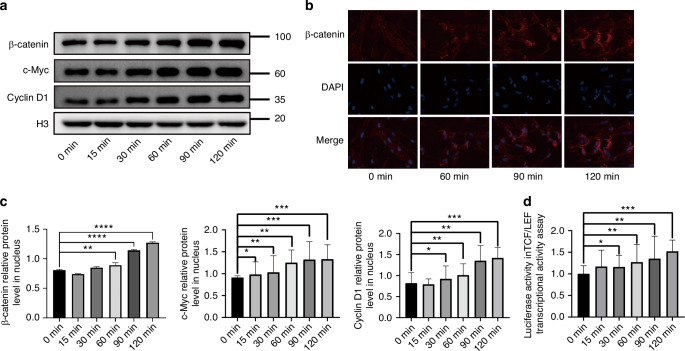
SLIT3 can reduce the degradation of β-catenin and promote its nuclear translocation. **a** Western blot analysis of β-catenin/c-Myc/Cyclin D1 in nucleus of SCAP after the addition of rhSLIT3, H3 normalized relative protein level; **b** Representative images of cyto-immunofluorescence staining of β-catenin in SCAP after the addition of rhSLIT3 for 60, 90 and 120 min (400×). Scale bars:100 μm. **c** Quantitative analysis of relative protein level of β-catenin/c-Myc/Cyclin D1 in nucleus of SCAP after the addition of rhSLIT3. *P < 0.05, **P < 0.01, ***P < 0.001, ****P < 0.000 1 versus the 0 min group (untreated baseline control) using one-way ANOVA followed by the Holm-Sidak post hoc multiple comparison test. **d** Luciferase assay for detecting TCF/LEF transcriptional activity of SCAP after the addition of rhSLIT3. *P < 0.05, **P < 0.01, ***P < 0.001, ****P < 0.000 1 versus the 0 min group (untreated baseline control) using one-way ANOVA followed by the Holm-Sidak post hoc multiple comparison test

Given that inactivation of GSK3β is the canonical signal for preventing β-catenin degradation, we next hypothesized that SLIT3 treatment would lead to an accumulation and nuclear translocation of β-catenin. As expected, Western blot analysis of nuclear fractions showed a time-dependent increase in nuclear β-catenin levels, which peaked at 90–120 min following rhSLIT3 stimulation (Fig. 7a, c). This was accompanied by a corresponding increase in the abundance of the canonical β-catenin transcriptional targets, c-Myc and Cyclin D1. Immunofluorescence staining visually confirmed a progressive accumulation of β-catenin within the nucleus (Fig. 7b). Concordantly, a TCF/LEF luciferase reporter assay showed that SLIT3 stimulation significantly increased β-catenin-dependent transcriptional activity (Fig. 7d). These findings establish a functional link between SLIT3 stimulation and the activation of β-catenin-mediated transcription.

**Fig. 8:**
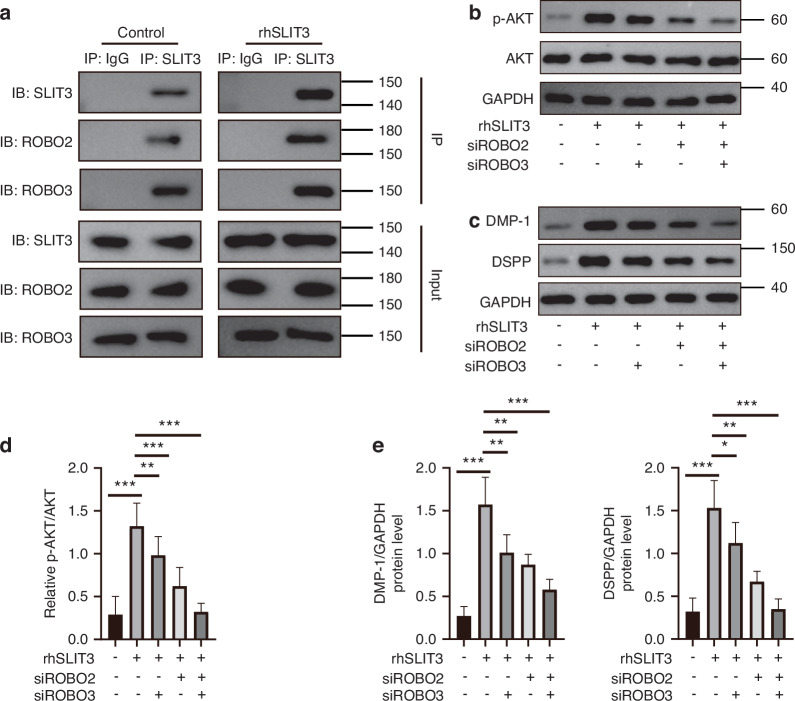
SLIT3 activates the Akt/GSK3β/β-catenin signaling pathway in SCAP through ROBO2/ROBO3 receptors. **a** Protein immunoprecipitation of SLIT3 from SCAP confirmed the presence of ROBO2 and ROBO3, with rhSLIT3 treatment further enhancing the co-immunoprecipitated levels of ROBO2 and ROBO3, indicating a stronger interaction. **b** Western blot analysis of Akt phosphorylation following siRNA-mediated knockdown of ROBO2/ROBO3 in SCAP. **c** Western blot analysis of DMP1/DSPP following siRNA-mediated knockdown of ROBO2/ROBO3 in SCAP. **d** Quantitative analysis of (**b**). **P < 0.01, ***P < 0.001 versus the rhSLIT3 group using one-way ANOVA followed by the Holm-Sidak post hoc multiple comparison test. **e** Quantitative analysis of (**c**). *P < 0.05, **P < 0.01, ***P < 0.001 versus the rhSLIT3 group using one-way ANOVA followed by the Holm-Sidak post hoc multiple comparison test

Having observed that SLIT3 modulates these intracellular signaling components, we then sought to identify the upstream cell-surface receptors responsible for initiating these events. Based on bioinformatics analysis showing Slit3 co-expression with Robo2 and Robo3 in vivo (Supplementary Fig. S2), we hypothesized that ROBO2 and/or ROBO3 were the functional receptors in SCAP. Co-immunoprecipitation (Co-IP) assays confirmed a direct physical interaction, as both ROBO2 and ROBO3 were pulled down with SLIT3, an interaction that was enhanced by rhSLIT3 treatment (Fig. 8a). To validate their functional necessity, we used siRNA to knock down Robo2 and Robo3. This knockdown completely abolished the ability of rhSLIT3 to induce Akt phosphorylation (Fig. 8b, d) and to upregulate the odontogenic markers DMP-1 and DSPP (Fig. 8c, e). These data demonstrate that SLIT3 requires ROBO2/ROBO3 receptors to modulate Akt activity and promote odontogenic differentiation.

**Fig. 9:**
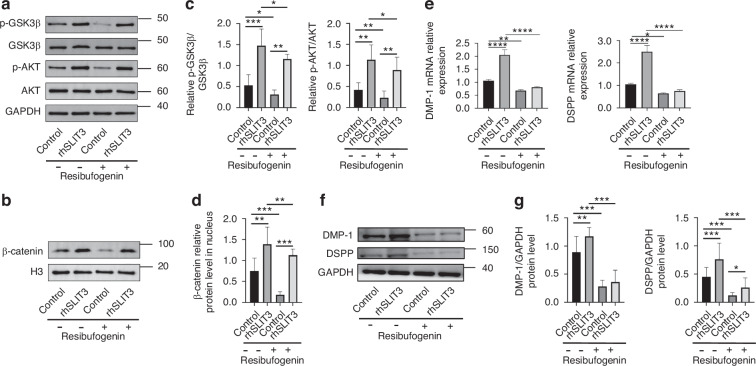
The promotion of SLIT3 on odontogenic differentiation of SCAP were canceled after inhibition of Akt/GSK3β/β-catenin signaling pathway. **a**, **b** Western blot for Resibufogenin inhibition efficiency test of Akt/GSK3β/β-catenin signaling pathway. **c**, **d** Quantitative analysis of **a**, **b**. *P < 0.05, **P < 0.01, ****P < 0.000 1 versus the control group without Resibufogenin using one-way ANOVA followed by the Holm-Sidak post hoc multiple comparison test. **e** RT-PCR analysis of DMP-1 and DSPP mRNA expression in SCAP after blocking Akt/GSK3β/β-catenin signaling pathway and/or adding rhSLIT3. **f** Western blot analysis of DMP-1 and DSPP in SCAP after blocking Akt/GSK3β/β-catenin signaling pathway and/or adding rhSLIT3; **g** Quantitative analysis of (**f**). *P < 0.05, **P < 0.01, ****P < 0.000 1 versus the control group without Resibufogenin using one-way ANOVA followed by the Holm-Sidak post hoc multiple comparison test

Finally, to determine if the observed modulation of these signaling proteins is functionally required for SLIT3-mediated odontogenesis, we employed Resibufogenin, a pharmacological inhibitor known to disrupt these signaling events.^22^ As confirmed in Fig. 9a–d, Resibufogenin pre-treatment blocked the rhSLIT3-induced phosphorylation of Akt and GSK3β. Crucially, in the presence of this inhibitor, rhSLIT3 completely failed to upregulate the expression of the odontogenic markers DMP-1 and DSPP (Fig. 9e–g). This result confirms that the pro-odontogenic function of SLIT3 is dependent on the activity of these signaling intermediates.

**Fig. 10:**
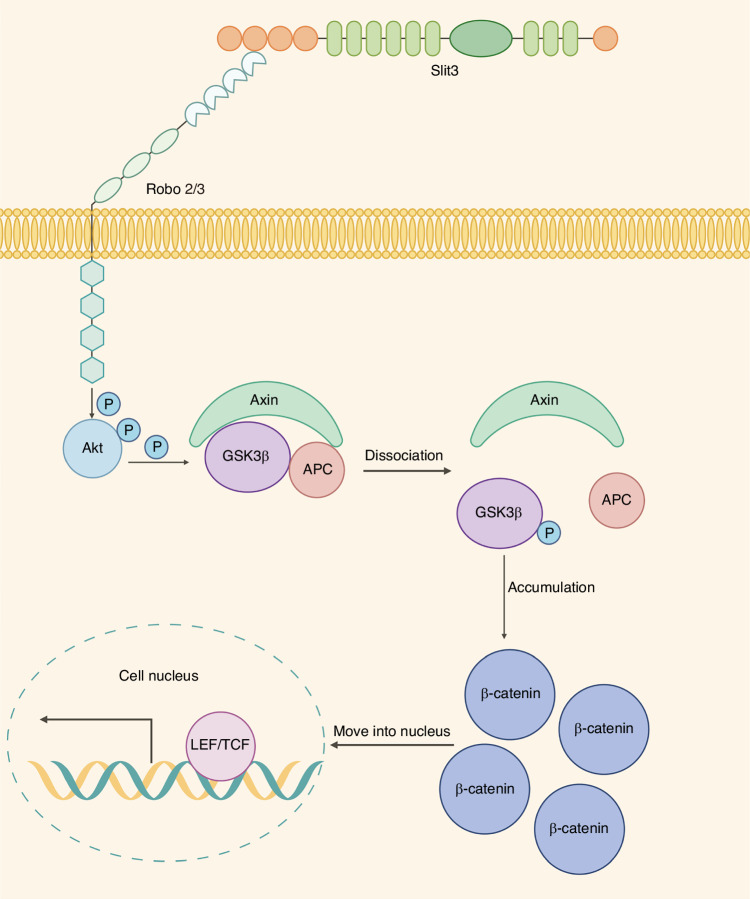
Schematic diagram of SLIT3 regulating odontogenic differentiation of SCAP via the Akt/GSK3β/β-catenin pathway

In summary, our results delineate a novel regulatory mechanism (Fig. 10) where SLIT3, acting via ROBO2/3 receptors, modulates the phosphorylation state and activity of Akt/GSK3β/β-catenin signaling module to drive the odontogenic differentiation of SCAP.

## Discussion

As structural proteins, extracellular matrix proteins can regulate the activities of growth factors and proteases by interacting with receptors and integrins on the cell membrane,^23^ which plays an important role in dentin formation and mineralization. Extracellular matrix protein SLIT3 initially entered the vision of researchers as guidance for axonal development.^24^ With the gradual in-depth and clarified study of SLIT3 in the field of neurodevelopment, many researchers began to explore the role of SLIT3 in other life activities. So far, SLIT3 has been found to be involved in angiogenesis, tumorigenesis, inflammation, regulation of bone metabolism and so on.^25,26^ Some studies have found that SLIT3 is a new coupling factor of bone metabolism, which can inhibit the migration and fusion of pre-osteoclasts so that inhibiting bone resorption; it can also promote the proliferation and migration of osteoblasts, and even indirectly promote bone formation by promoting angiogenic factors.^17,27^

Bone and teeth are important hard tissue organs, and they have many similarities in the process of formation. For example, bone morphogenetic protein (BMP), osterix (OSX) and bone sialoprotein (BSP) which play important roles in bone metabolism have also been proved to play essential roles in tooth development.^28–30^ However, there are few reports on the role of bone metabolic coupling factor SLIT3 in tooth development. At present, it has only been reported that the expression of RNA of Slit family (Slit1-Slit3) during the development of tooth in vivo.^31^ RNA of *Slit3* is mainly expressed in the mesenchymal components of the tooth germ, including dental papilla and dental follicle, and lasts from the bud stage to the bell stage.

One of the most intriguing findings of our study is the apparent spatial disparity between *Slit3* mRNA expression and the localization of its protein product, which points to a complex intercellular signaling network. Our re-analysis of both public scRNA-seq data and our own microarray dataset consistently demonstrated that *Slit3* transcripts are predominantly expressed in the mesenchymal components of the tooth germ (dental papilla and odontoblasts), with negligible expression in the epithelium. Conversely, our immunohistochemical analysis revealed a prominent SLIT3 protein signal not only on mesenchymal odontoblasts but also strongly on epithelial-derived ameloblasts, where the mRNA is absent. We propose that this is not a contradiction but rather compelling evidence for a dual signaling mechanism. The strong protein signal on ameloblasts is best explained by a paracrine signaling axis. As a canonical secreted ligand, it is highly plausible that SLIT3 is synthesized by dental mesenchymal cells, secreted into the extracellular matrix, and then diffuses to bind its cognate receptors on the surface of adjacent ameloblasts.^26^ This accumulation of the ligand on its target cell is a classic feature of paracrine signaling and would lead to the strong immunostaining signal observed, a principle fundamentally established in the context of axon guidance where midline glia-secreted SLIT repels ROBO-expressing neurons.^24^ Furthermore, our study’s central focus investigates the second mechanism: an autocrine/paracrine signaling axis within the mesenchyme itself. Our re-analysis of the scRNA-seq data provides critical support for this, confirming that the canonical receptors, Robo2 and Robo3, are robustly expressed in the same mesenchymal cell populations that express *Slit3*. This co-expression of ligand and receptors provides a strong in vivo basis for the autocrine/paracrine signaling loop that we investigated in our SCAP model, a mechanism that has also been described in other systems, such as the positive feedback loop of SLIT3 in osteoclasts during bone remodeling.^32^ Therefore, by integrating transcriptomic and proteomic data, we paint a more dynamic picture: mesenchymal-derived SLIT3 likely participates in both a mesenchyme-epithelium paracrine axis influencing ameloblasts and a mesenchymal autocrine/paracrine axis that regulates odontoblast differentiation—the latter being the core mechanism elucidated in this study, and both being consistent with the established role of the SLIT/ROBO system in mediating complex intercellular communication.^26^

Stem cells from apical papilla come from the undeveloped dental papilla tissue in the apical region, and have the potential of multi-directional differentiation, such as osteogenesis, odontogenesis, angiogenesis and adipogenesis.^33^ Compared with dental pulp stem cells, SCAP have better proliferation efficiency, migration ability and differentiation versatility, so it is an ideal cell model for studying tooth development.^34^ Our initial experiments immediately validated this choice: we found that, consistent with our findings in developing mouse molars, the expression of both SLIT3 mRNA and protein significantly increased during the odontogenic differentiation of SCAP in vitro. This powerful congruency between in vivo and in vitro data confirms the fundamental role of SLIT3 in dentinogenesis. Our use of SCAP isolated from human immature third molars was a deliberate choice to enhance the study’s translational impact, aligning with the gold-standard model for such investigations.^8^ This approach also pragmatically addresses the challenge of obtaining sufficient cell yields for extensive molecular assays, which is technically prohibitive with neonatal mouse molars. While we acknowledge that the close anatomical proximity of the apical papilla to the apical follicle creates a potential for minor cross-contamination during micro-dissection, the functional signature of our cell population is unequivocal. The robust induction of definitive odontogenic markers, particularly DSPP and DMP-1, provides compelling functional evidence that our cultures behave as a homogenous odontoprogenitor pool. This validation provides a solid foundation as we move to dissect the specific regulatory role of SLIT3.

However, the regulatory role of SLIT3 on odontogenic differentiation of SCAP and its mechanism are still unclear. Therefore, in our study, we induced SCAP with low/high expression of SLIT3 to odontoblasts in vitro and found that the proliferation and odontogenic differentiation ability of SCAP were positively correlated with the expression of SLIT3. In the process, we also found an interesting phenomenon: the SLIT3 expression increased in mRNA level by rhSLIT3 treatment (Supplementary Fig. S1d). Through the literature review, we found that SLIT3 secreted by osteoclasts could act on membrane receptors through autocrine, and then promoted the mRNA expression of Slit3 through the mediate of Rac1 GTPase, forming a positive feedback cycle.^32^ Therefore, we speculated whether SLIT3 in SCAP also had a positive feedback loop similar to that in osteoclasts. On the other hand, our experimental data showed that the mRNA expression of SLIT3 increased during the odontogenic differentiation of SCAP (Fig. 2e). At the same time, the addition of rhSLIT3 promoted the odontogenic differentiation of SCAP (Fig. 4a–g). These results also explained the increased mRNA expression of SLIT3 in the rhSLIT3 group to some extent. Of course, more data are needed to prove this conjecture.

In our study, we qualitatively detected the mineralization ability of SCAP after the change of SLIT3 by alkaline phosphatase staining and alizarin red staining. However, due to the rich and diverse differentiation potential of SCAP, it is also possible to differentiate into osteoblasts under the condition of mineralization induction. ALP staining and ARS staining cannot accurately indicate the enhancement of osteogenic differentiation or odontogenic differentiation of SCAP. DMP-1 and DSPP are highly phosphorylated proteins belonging to the small integrin-binding ligand N-linked glycoproteins (SIBLINGs) family, which are recognized positive regulatory factors of dentin mineralization, playing an indispensable role in the secretion and mineralization of dentin.^35^ DSPP is primarily specifically expressed in odontoblasts, and many studies have used it as a specific marker for detecting odontogenic differentiation or odontoblasts.^36,37^ DMP-1 also has certain specificity in its expression during the early and late stages of odontoblast differentiation, and it is one of the common markers used to detect odontogenic differentiation of stem cells, in addition to DSPP.^38,39^ Therefore, we further selected DMP-1 and DSPP as markers of odontogenic differentiation of SCAP and found that SLIT3 could indeed promote the expression of DMP-1 and DSPP in SCAP, which was consistent with previous results.

SLIT3, a secretory protein, functions by binding to ROBO receptors, which transduce signals to intracellular effectors. The SLIT/ROBO signaling network is complex and context-dependent, but studies of SLIT2, a close homolog of SLIT3, offer a critical clue. The interaction between SLIT2 and ROBO modulates the activity of the central kinase Akt.^26^ Based on this precedent and the structural similarity within the SLIT family, we hypothesized that SLIT3 may also converge on the Akt signaling pathway in SCAP. Our study confirmed this hypothesis: exogenous SLIT3 stimulation rapidly increased Akt phosphorylation in SCAP, demonstrating SLIT3’s ability to activate Akt signaling in this context. The activation of Akt establishes a direct mechanistic link to another pathway crucial for odontogenesis. Akt is a recognized upstream inhibitor of GSK3β, a pivotal kinase that targets β-catenin for degradation.^40–42^ The classical Wnt/β-catenin signaling pathway plays a crucial role in embryonic development and postnatal growth and is evolutionarily conserved.^43^ Extensive research indicates the role of Wnt/β-catenin in tooth morphogenesis and dental tissue formation.^44^ The Wnt/β-catenin signaling pathway positively influences the proliferation and differentiation of SCAP and is a key regulator of odontoblast differentiation.^45,46^

While it remained conceivable that Akt, GSK3β, and β-catenin could act as independent downstream targets of SLIT3, compelling evidence accumulated from extensive literature has firmly established their participation in a well-established hierarchical cascade.^47–50^ Specifically, activated Akt phosphorylates GSK3β at Ser9, resulting in its inactivation. In its active form, GSK3β participates in the β-catenin destruction complex (comprising CK1, APC, and Axin), which phosphorylates β-catenin to trigger its ubiquitination and proteasomal degradation. When GSK3β is inhibited by Akt-mediated phosphorylation, β-catenin becomes stabilized, accumulates in the cytoplasm, and translocates to the nucleus to regulate transcription of target genes. Given the strength of this canonical mechanistic framework, we reasoned that re-demonstrating these established molecular relationships would be redundant and focused instead on verifying their activation status within our experimental model. Thus, we propose that SLIT3 exerts its pro-odontogenic effects through the Akt/GSK3β/β-catenin signaling cascade module.

Our subsequent experiments systematically validated this proposed cascade. We first demonstrated that SLIT3 not only activated Akt but also induced the inhibitory phosphorylation of its downstream target, GSK3β. This led to the predicted outcome: inhibited degradation and subsequent nuclear translocation of β-catenin, accompanied by the activation of its transcriptional targets. Crucially, when this pathway was pharmacologically blocked using the inhibitor Resibufogenin, the pro-odontogenic effects of SLIT3 were completely abrogated. Taken together, these results provide strong evidence for a novel signaling axis in which SLIT3 promotes odontogenic differentiation of SCAP by activating the Akt/GSK3β/β-catenin signaling module. While our data primarily reflect correlative activation patterns among these molecules, the hierarchical relationship within this cascade has been conclusively demonstrated in multiple investigations.^47–50^ Therefore, our schematic (Fig. 10) represents this well-recognized signaling hierarchy rather than a speculative model. In future work, we plan to further delineate the upstream molecular events linking SLIT3–ROBO2/3 to the Akt–GSK3β–β-catenin axis through targeted mechanistic assays such as co-immunoprecipitation or pathway inhibition, to refine our understanding of SLIT3–ROBO signaling in odontogenic regulation.

After transfer into the nucleus, β-catenin can interact with TCF/LEF and transcriptional coactivators that regulate the expression of some target genes to form a complex to activate the transcription of downstream target genes.^51^ The biological behavior of β-catenin after nuclear translocation in SCAP and the effect changes at the end of the signal pathway are still lack of exploration and verification of related experiments in this study, which will be a direction of follow-up research. In addition, SLIT3/ROBO can promote the proliferation and migration of osteoblasts by regulating the activity of cytoplasmic kinase Abl and then regulating the activity of downstream β-catenin.^52^ So, is it possible for SLIT3 to mediate the change of β-catenin through Abl in SCAP, and then regulate the odontogenic differentiation? This is also a problem worthy of future exploration.

In summary, our study discovered and explored the role of SLIT3 in tooth development and dentin formation for the first time, further improved the signal regulation network of dentin formation, helped us to have a more comprehensive understanding of the process of dentin formation and its influencing factors, and provided a new theoretical basis and treatment ideas for dentin defect repair and regeneration.

## Materials and methods

### Re-analysis of single-cell RNA sequencing and microarray datasets

We obtained single-cell RNA sequencing data from the Gene Expression Omnibus (GEO) dataset GSE189381, which pertains to the digestion of teeth and surrounding tissue at five distinct developmental stages (E13.5, E14.5, E16.5, PN3.5, and PN7.5). To analyze the raw read counts at each stage, we utilized the R package Seurat (Ver. 4.0.5) for downstream single-cell level analyses. Cells expressing genes in fewer than 3 cells, mitochondrial genes constituting more than 50% of the expression, and cells with fewer than 200 genes were filtered out. Cell cycle information was obtained for each cell using the “CellCycleScoring” method. To increase interpretability, the gene expression matrixes were transformed using the “SCTransform” function in Seurat, with consideration of 4 000 highly variable genes. We applied regressions to address the impact of mitochondrial genes and cell cycles, followed by batch effect correction and dataset integration using the “FindIntegrationAnchors” and “IntegrateData” functions. Subsequently, the “FindNeighbors” and “FindClusters” functions were employed, leveraging unsupervised clustering based on the first 50 principal components with a resolution of 1. Uniform manifold approximation and projection (UMAP) plots were generated for non-linear dimensional reduction visualization. Differentially expressed genes (DEGs) were determined using the “FindAllMarkers” function with the ‘MAST’ package (Ver. 1.20.0). The dental mesenchymal cell subclusters and the epithelial cell subcluster were annotated at different developmental stages based on the DEGs and previously reported markers for the dental mesenchyme.

We conducted a reanalysis of a microarray dataset on mouse dental epithelial, dental papilla, and dental follicle cells groups using the same workflow outlined in our prior study.^53^ Our investigation specifically emphasized and visualized the expression level of SLIT3 in different groups.

### Animal models and Immunohistochemical analysis

For the mandibular molar developmental model, C57BL/6 mice were obtained from Dashuo Experimental Animals Co., Ltd., Chengdu, China. The Ethics Committee of West China Hospital of Stomatology approved all procedures. Mice at postnatal (PN) 1, 7, 14, and 21 were harvested. Mandibles were dissected, fixed in 4% paraformaldehyde at 4 °C overnight, and then decalcified in 12.5% DEPC-treated EDTA (pH 7.4) for 1–4 weeks based on age. Decalcified tissues were dehydrated, paraffin-embedded, and 5-µm thick sections were prepared. Immunohistochemical examination using standard procedures reviewed molecular expression. Polyclonal rabbit anti-SLIT3 (1:100, ab198726, Abcam, Cambridge, UK) served as primary antibodies, and PBS was used in the negative control group. Immune complexes were visualized using a DAB kit (Zhongshan Golden Bridge Biotechnology, Beijing, China).

For the SCAP/β-TCP ectopic transplantation model, 6-week-old female nude mice were sourced from Gempharmatech Co., Ltd., Chengdu, China. The Ethics Committee of West China Hospital of Stomatology approved the procedures. β-TCP Scaffold (Materials Manufacturing Core, Sichuan University, Chengdu, China) and SCAP with siSLIT3/rhSLIT3 or their controls were prepared as described previously to form the β-TCP block and cell complexes.^54,55^ After anesthesia with 1.2% tribromoethanol (0.2 mL per 10 g), the complexes were implanted into subcutaneous pockets on both sides of the mice’s back. Six weeks post-transplantation, mice were euthanized with an overdose of ketamine-xylazine (Ketamine 300–360 mg/kg + xylazine 30–40 mg/kg, IP) following cervical dislocation once they reached a surgical plane of anesthesia verified by a toe pinch test. Ectopic transplants were collected, fixed in 10% buffered formaldehyde, decalcified in 12.5% DEPC-treated EDTA for 1 week, paraffin-embedded, and 5-µm thick sections were prepared and stained with Hematoxylin-Eosin stains (Solarbio, Wuhan, China) per the manufacturer’s protocol. Polyclonal rabbit anti-SLIT3 (1:100, ab198726, Abcam, Cambridge, UK), anti-DSPP (1:1 000, Novus Biologicals, Colorado, USA), and anti-DMP-1 (1:1 000, Novus Biologicals, Colorado, USA) were used as primary antibodies for immunohistochemical examination, and PBS was used in the negative control group. Immune complexes were visualized using a DAB kit (Zhongshan Golden Bridge Biotechnology, Beijing, China). Expression levels of DSPP and DMP-1 were semi-quantitatively classified based on the immunoreactive H-score (range 0–300), calculated as the product of intensity score (1, faint/weak; 2, moderate; 3, strong) and distribution score (percentage from 1 to 100).

### Cell isolation and culture

This research was approved by the Ethics Committee of West China Hospital of Stomatology, and informed consent was obtained from all patients. Human third molars with immature roots were collected from healthy patients 16–20 years old at the oral surgery department of West China Hospital of Stomatology, Sichuan University, Chengdu, China. Briefly, the apical papilla was separated from the immature roots and minced. Then, the tissues were digested in a mixed solution containing 3 mg/mL collagenase type I (Sigma-Aldrich, MO, USA) and 4 mg/mL dispase II (Sigma-Aldrich, MO, USA) for 30 min at 37 °C. Cells were cultured in growth medium including α-modification of Eagle medium (α-MEM; Gibco, MD, USA) supplemented with 20% fetal bovine serum (Gibco, MD, USA), 100 U/mL penicillin, and 100 mg/mL streptomycin (Gibco, MD, USA) at 37 °C in 5% CO_2_. We used the third passage of cells in the following experiments.

### Odontogenic differentiation induction

For odontogenic differentiation, SCAP were seeded in 6-well plates at a density of 1 × 10^5^per well. When reaching 70%–80% confluence, SCAP were cultured in odontogenic differentiation induction medium containing α-MEM, 10% FBS, 50 mg/ml ascorbic acid, 10 nmol/L dexamethasone and 10 mM β-glycerophosphate (Sigma-Aldrich, MO, USA). The mineralization ability of SCAP were evaluated after 1–2 weeks induction.

### Down-regulation of SLIT3 and ROBO2/3 and Up-regulation of SLIT3

To downregulate SLIT3 expression in SCAP cells, the standard culture medium was first replaced with serum-free medium to induce a starvation state prior to transfection. Subsequently, SLIT3-specific siRNA was transfected using Lipofectamine 2000 (Thermo Fisher Scientific, MA, USA), while a non-targeting siRNA served as the negative control. Unless otherwise specified, siROBO2 and siROBO3 were transfected following the same protocol. After incubation for 6–8 h, the transfection reagent was replaced with the ordinary medium. For up-regulation of SLIT3 in SCAP, recombinant human SLIT3 protein was added into the medium at concentration of 0.5 μg/mL. The medium was renewed every 2 days. The change of expression of SLIT3 was detected by RT-PCR.

### Cell Counting Kit-8 Assay

The proliferation of SCAP with low/high expression of SLIT3 was assessed using a Cell Counting Kit-8 (CCK8; Dojindo, Kumamoto, Japan). In the CCK8 assay, 5 × 10^3^ SCAP per well were seeded in 96-well plates. CCK8(10 μL) solution was added to each well of the plate at different time points (days 1, 3, 5, 7, 10, and 14). According to the manufacturer’s instructions, the OD value was determined by measuring the absorbance at 450 nm, which represented the proliferation of SCAP by reflecting the change of cell number.

### Alkaline phosphatase (ALP) staining and Alizarin Red S (ARS) staining

SCAP were cultured in 24-well plates supplied with odontogenic differentiation induction medium. The medium was renewed every 2 days. For ALP staining, after 7 days of odontogenic induction, SCAP from each group were fixed with 4% Paraformaldehyde. Then, cells were stained with CIP/NBT Alkaline Phosphatase Color Development Kit (Beyotime, Shanghai, China). For ARS staining, after 14 days of odontogenic induction, SCAP from each group were fixed with 4% Paraformaldehyde. Then, cells were stained with 1% Alizarin Red S (Solarbio, Beijing, China).

### Quantitative real-time polymerase chain reaction

Total RNA of the SCAP were extracted by TRIzol reagent (TAKARA, Dalian, China) after 7, 10, or 14 days of odontogenic induction. RNA concentrations were measured using a Nanodrop Spectrophotometer (Thermo Fisher Scientific, MA, USA). Complementary DNA was synthesized from 1 000 ng RNA using a PrimeScript RT kit (TAKARA, Dalian, China). Real-time quantitative polymerase chain reaction was performed in a Roche 480 Light Cycler with SYBR Green Premix (Yeason, Shanghai, China). The program was set as follows: 40 cycles each involving 5 s of denaturation at 95 °C and 30 s of amplification at 60 °C. The mRNA expression levels of SLIT3, dentin sialophosphoprotein (DSPP) and dentin matrix protein 1 (DMP-1) were normalized to GAPDH using the 2^−ΔΔCT^ method. The primer sequences for RT-PCR are listed in Table 1.

**Table 1:** Primer sequences for quantitative real-time polymerase chain reaction

Gene	Forward primer sequence	Reverse primer sequence
*GAPDH*	GGAGCGAGATCCCTCCAAAAT	GGCTGTTGTCATACTTCTCATGG
*SLIT3*	CGGCATCACCGATGTGAAGAA	AGGCGCAGAGTTCGGATCT
*DMP-1*	CACTCAAGATTCAGGTGGCAG	TCTGAGATGCGAGACTTCCTAAA
*DSPP*	ATATTGAGGGCTGGAATGGGGA	TTTGTGGCTCCAGCATTGTCA

### Western blot analysis

Total proteins of the SCAP were extracted by Total Protein Extraction Kit (SAB, Maryland, USA) after 7, 10, or 14 days of odontogenic induction. The protein concentrations were determined by BCA protein assay reagent (Beyotime, Shanghai, China). Equal amounts of protein samples were loaded and separated by 10% sodium dodecyl sulfate polyacrylamide gel electrophoresis (SDS-PAGE) and transferred onto polyvinylidene fluoride (PVDF) membrane. After blocking with 5% BSA at room temperature for 1 h, the membranes were incubated at 4 °C overnight with primary antibodies against SLIT3 (1:500; R&D Systems, Minnesota, USA), DSPP (1:1 000; Novus Biologicals, Colorado, USA), DMP-1(1:500; Novus Biologicals, Colorado, USA) and GAPDH (1:5 000; Abcam, Cambridge, UK). The membranes were washed in TBST for three times, then they were incubated with the appropriate horseradish peroxidase conjugated secondary antibodies (1:10 000; Boster, Wuhan, China) at room temperature for 1 h. Finally, the bands were reacted with an enhanced chemiluminescence kit (Epizyme, Shanghai, China) and exposed to a Western blot analysis imaging system. Grayscale analysis was performed with Image J software.

### Extraction of nuclear protein

The nuclear proteins of the SCAP were obtained using the Nuclear Protein Extraction Kit (Beyotime, Shanghai, China). Briefly, the SCAP that have been treated with rhSLIT3 were harvested and dissociated in 200 μL Reagent A mixture containing 1 mmol/L PMSF (KeyGen BioTech, Nanjing, China). The cell suspension was vortexed for 5 s and incubated on ice for 15 min. Then, 10 μL Reagent B was added and the mixture was vortexed for 5 s following by 1 min ice bath and 5 s vortex again. After centrifugation at 12 000 × *g*, 4 °C for 5 min, the supernatant was collected as cytoplasmic protein and the precipitation was further resuspended in 50 μL nuclear protein extraction reagent containing 1 mmol/L PMSF. After being vortexed and ice bath in turn for 30 min, the mixture was centrifuged at 12 000 × *g*, 4 °C for 10 min and the supernatant was saved as nuclear protein.

### Akt/GSK3β/β-catenin signaling pathway validation

To make sure whether Akt/GSK3β/β-catenin signaling pathway were involved in the process, the total protein and nuclear protein of SCAP treated with 0.5 μg/mL recombinant human SLIT3 protein was extracted at 0, 15, 30, 60, 90, and 120 min respectively. The expression levels of p-Akt/Akt, p-GSK-3β/GSK-3β (1:1 000, all from Cell Signaling Technology) and β-catenin (1:5 000, Abcam, Cambridge, UK), c-Myc (1:1 000; Abcam, Cambridge, UK), Cyclin D1(1:1 000; Abcam, Cambridge, UK) in nucleus were detected by Western blot assay described previously. For these time-course experiments, the 0-min time point, collected immediately before the addition of rhSLIT3, served as the unstimulated baseline control for all comparisons. GAPDH (1:5 000; Abcam, Cambridge, UK) and H3 (1:1 000; Abcam, Cambridge, UK) were selected as loading control protein in total protein and nuclear protein respectively. Thereafter, the Akt/GSK3β/β-catenin inhibitor Resibufogenin (inhibitor of Akt, GSK-3β and β-catenin, 4 μM) (Selleck, Houston, TX) was added to the culture medium. The mRNA and protein expression of DSPP and DMP-1 of rhSLIT3-treated SCAP were examined to evaluate the odontogenic differentiation capacity.

### TCF/LEF transcriptional activity assay

To assess TCF/LEF transcriptional activity, SCAP cells were transfected with the TOPFlash luciferase reporter plasmid (Beyotime, Shanghai, China), which provides high sensitivity for detecting Wnt/β-catenin signaling. Following transfection, the cells were treated with recombinant human SLIT3 (rhSLIT3), and Wnt pathway activity was evaluated at designated time points using the Dual-Luciferase Reporter Assay System. Firefly luciferase activity was normalized to Renilla luciferase to control for transfection efficiency.

### Immunofluorescence staining

For immunofluorescence analysis, cells were seeded on slides. After washing and fixation in 4% paraformaldehyde, the slides were incubated in β-catenin (1:250, Abcam, Cambridge, UK) antibodies at 4 °C overnight. After 1 h of incubation with Goat anti-Rabbit secondary antibody (1:1 000; Beyotime, Shanghai, China) at 37 °C in the dark, 4,6-diamidino-2-phenylindole (DAPI) was used to stain the nucleus. Finally, the slides were observed under a fluorescence microscope (Leica, Wetzlar, Germany) and captured.

### Statistical analysis

All experiments were independently performed at least three times. Data are presented as the mean ± standard deviation. Differences between groups were evaluated by Student’s *t*-test or one-way analysis of variance test using SPSS 24.0 software (SPSS Inc., Chicago, USA). *P* < 0.05 was considered statistically significant.

## Supplementary information

Supplementary information
