# Supplementary material for: SLIT3: a novel regulator of odontogenic differentiation through Akt/GSK3β/β-catenin signaling pathway
Source: Int J Oral Sci. 2026 Apr 13;18:35. doi: 10.1038/s41368-026-00426-7 (PMC13071080; doi:10.1038/s41368-026-00426-7)
Supplement: Supplementary file 1 — Supplementary information [file 41368_2026_426_MOESM1_ESM.docx]

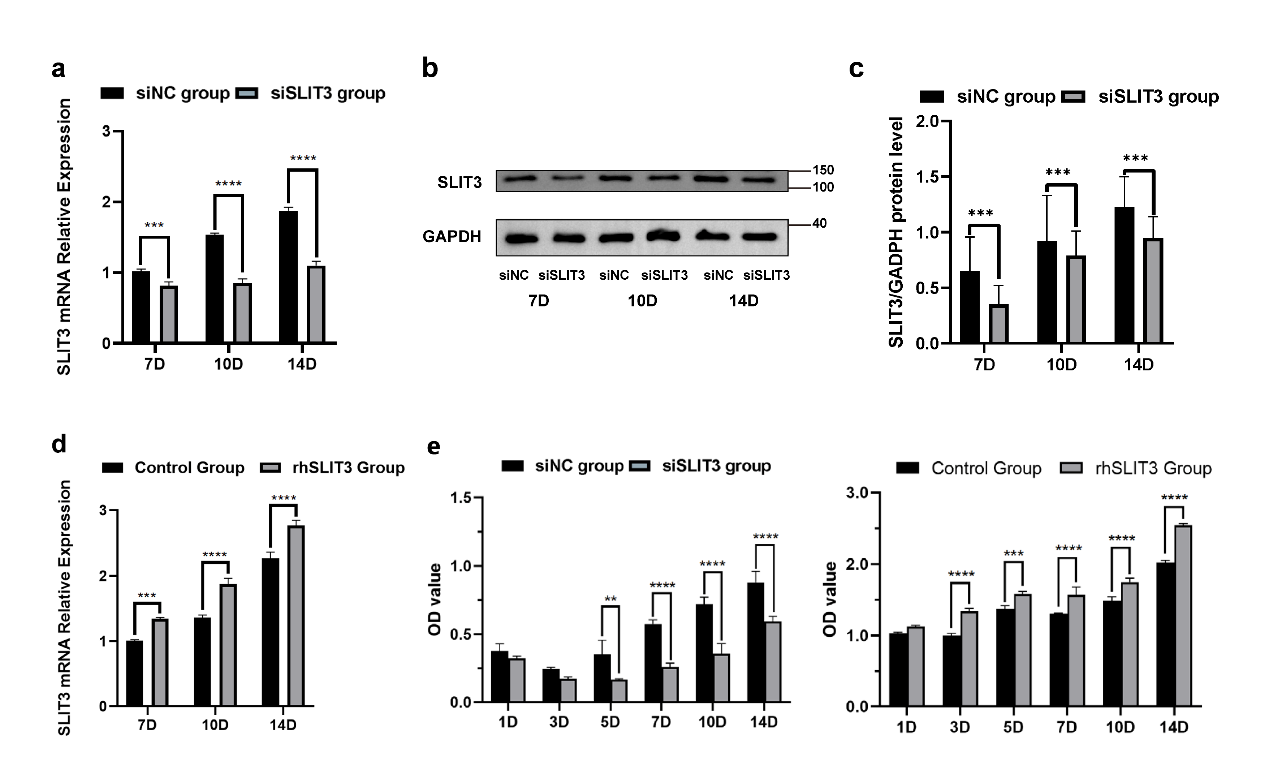
**Supplementary Fig.S1:** The expression of SLIT3 is positively correlated with the proliferation of SCAP. (a) RT-PCR analysis of SLIT3 mRNA expression in siNC group and siSLIT3 group. ***P＜0.001, ****P＜0.0001 versus the siNC group using two-way ANOVA followed by the Holm‐Sidak post hoc multiple comparison test. (b) Western blot for SLIT3 expression in siNC and siSLIT3 groups in undifferentiated SCAPs. (c) Quantitative analysis of (b). ***P＜0.001 versus the siNC group using two-way ANOVA followed by the Holm‐Sidak post hoc multiple comparison test. (d) RT-PCR analysis of SLIT3 mRNA expression in control group and rhSLIT3 group. ***P＜0.001, ****P＜0.0001 versus the control group using two-way ANOVA followed by the Holm‐Sidak post hoc multiple comparison test. (e) The OD value of siNC group and siSLIT3 group in CCK8 assay, and the OD value of control group and rhSLIT3 group in CCK8 assay. ***P＜0.001, ****P＜0.0001 versus the control group using two-way ANOVA followed by the Holm‐Sidak post hoc multiple comparison test.


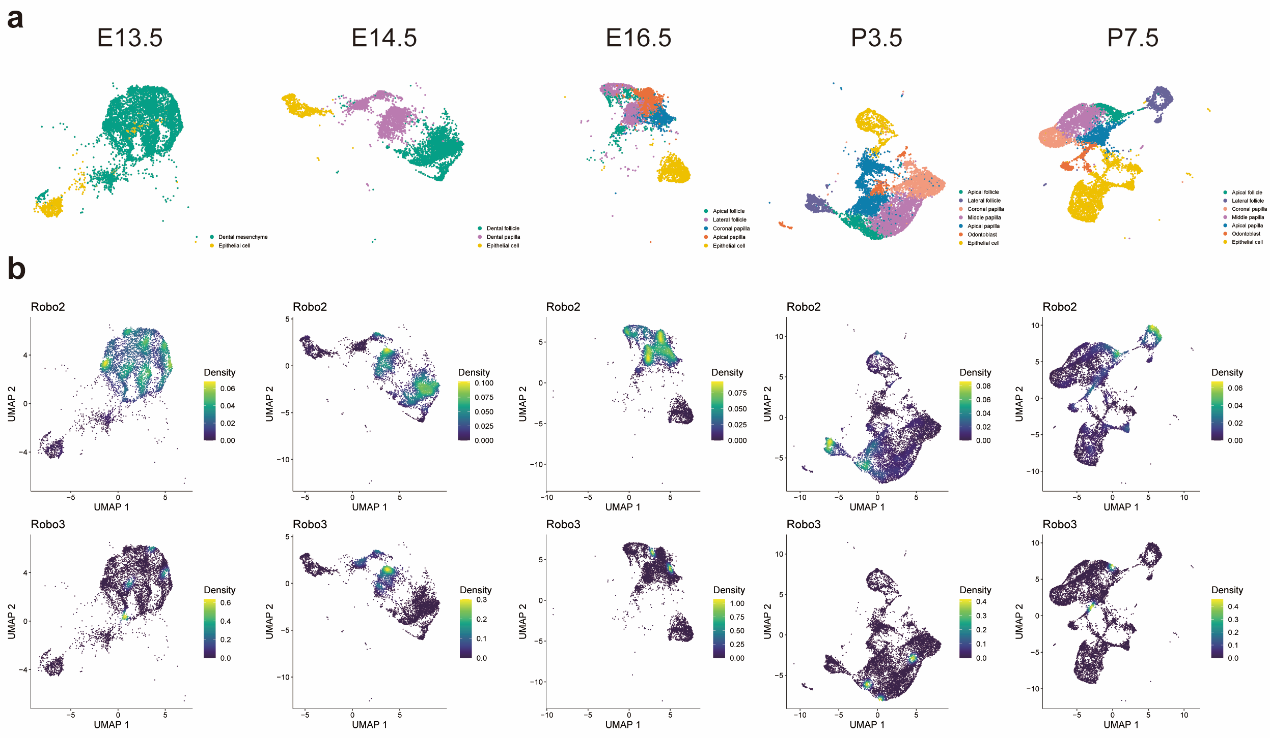


**Supplementary Fig.S2:** In vivo expression of Robo2 and Robo3 during mouse tooth development. (a) UMAP plots illustrating single-cell data from embryos to postnatal stages in the tooth and surrounding tissues; (b) Expression of Robo2 and Robo3 at different embryonic and postnatal stages, with dotted lines indicating mesenchymal cell subpopulations.
